# Research on Infant Health Diagnosis and Intelligence Development Based on Machine Learning and Health Information Statistics

**DOI:** 10.3389/fpubh.2022.846598

**Published:** 2022-06-02

**Authors:** Siyu Wang, Min Li, Soo Boon Ng

**Affiliations:** ^1^Teachers College, Chengdu University, Chengdu, China; ^2^SEGI University, Petaling Jaya, Malaysia

**Keywords:** machine learning, health information statistics, infant health diagnosis, intelligence development, big data

## Abstract

Intelligent health diagnosis for young children aims at maintaining and promoting the healthy development of young children, aiming to make young children have a healthy state and provide a better future for their physical and mental health development. The biological basis of intelligence is the structure and function of human brain and the key to improve the intelligence level of infants is to improve the quality of brain development, especially the early development of brain. Based on machine learning and health information statistics, this paper studies the development of infant health diagnosis and intelligence, physical and mental health. Pre-process the sample data, and use the filtering method based on machine learning and health information statistics for feature screening. Compared with traditional statistical methods, machine learning and health information statistical methods can better obtain the hidden information in the big data of children's physical and mental health development, and have better learning ability and generalization ability. The machine learning theory is used to analyze and mine the infant's health diagnosis and intelligence development, establish a health state model, and intuitively show people the health status of their infant's physical and mental health development by means of data. Moreover, the accumulation of these big data is very important in the field of medical and health research driven by big data.

## Introduction

In the process of improving people's quality of life, China pays more and more attention to the healthy development of human beings. Although people have deepened their understanding of human health under the promotion of this information age, China's research in the field of children's physical and mental health development still needs to be further deepened (1). Intelligence, also known as intelligence, refers to the people's response and processing ability to the surrounding environment and things. Human beings have extremely high intelligence, which is characterized by observation ability, memory ability, and thinking ability. Children's health diagnosis and intellectual development are extremely important factors during their childhood and even in their future life. Only when children grow up healthily, they will get good development in the future (2). In the field of children's health, diagnosis of children's intelligent health is the top priority. Children's intelligent health diagnosis aims to maintain and promote the children's healthy development, and is determined to let children have a good state of health and provide a better future for the children's physical and mental health development (3). The biological basis of intelligence is the structure and function of human brain. The key to improve the intelligence level of infants is to improve the quality of brain development, especially the early development of brain. Children's physical and mental health development inspectors are still in the manual stage of processing the children's physical condition data, which not only increases the workload of children's physical condition inspectors, but also may delay the treatment of children's condition (4). In the great project of improving population quality and cultivating socialist successors, the cultivation of infants' health diagnosis and intellectual development ability is a key link that cannot be ignored. It is the basic project to improve the children's physical and mental health development. This foundation must be laid well and firmly. With the rapid progress and development of information technology, various industries have accumulated a large amount of information and data. How to mine valuable information from these data is a problem that all fields need to face (5). As one of the main methods of data analysis, machine learning is widely used in data mining.

Based on machine learning and health information statistics, this paper studies the development of infant health diagnosis and intelligence, physical and mental health. Pre-process the sample data and use the filtering method based on machine learning and health information statistics for feature screening. Finally, using multiple machine learning algorithms, from single classifier, integrated classifier to fusion classifier, the advantages and disadvantages of multiple machine learning models are systematically explored to improve the prediction accuracy and fitting effect of the models (6, 7). Compared with traditional statistical methods, machine learning and health information statistical methods can better obtain the hidden information in the big data of children's physical and mental health development, and have a better learning ability and generalization ability (8). Machine learning is the study of making computers to imitate the way of human learning and thinking, and making computers have the ability of self-learning. Through learning, the computer can constantly update and reorganize the existing cognitive style, so that its performance can be continuously improved. Machine learning is the core of the development of artificial intelligence and the fundamental way to make inanimate computers intelligent. Machine learning is a process in which computers can identify and acquire knowledge through programming language and mathematical principles, and continuously improve the performance of infant health diagnosis and intelligence development, which can be roughly divided into supervised learning and unsupervised learning. The self-learning ability of machine learning is very important for data analysis, and it is of great significance for regression prediction and classification problems. The regression function can play a great role in finance, economic market, transportation, and benefit management. The core idea of machine learning is not to design a specific learning mode to let the machine learning algorithm do it, but to let the machine learning algorithm learn the laws and essential characteristics of data by itself. There are two kinds of unsupervised learning ideas. The first unsupervised learning idea is that when the training network parameters don't specify accurate data set classification, it adopts a weighted incentive and punishment system, which can increase and decrease the size of network parameters in the network. Supervised learning establishes a prediction model by analyzing the logical relationship between features and target variables, so as to obtain the prediction results of unknown data (9). Unsupervised learning analyzes the logical relationship of sample data without objective variables. Clustering analysis and association rule analysis are typical unsupervised learning.

When there is category imbalance in the development of physical and mental health in the health information statistical dataset of machine learning, it will have a great impact on the classification sensitivity of machine learning (10). In fact, in the real world, the data are unbalanced. Class imbalance problems are widely used in many aspects. In the use of all descriptions, a few classes have high sensitivity, and its information is the target. In the big data technology based on machine learning and health information statistics; massive data sources are a necessary requirement for big data analysis (11). Based on the machine learning and health information statistics, newborn should be trained to crawl and walk on time, on the basis of child's health diagnosis of hand movements. Training child to crawl can expand the range of child's activities, enable them to move freely, and make efforts to get in touch with interested objects, which have a positive effect on the development of perception and intelligence. The healthcare for toddler around 1 year old is mainly to gradually train to stand and walk. Only with the data analysis method supported by sufficient data can we provide a good personalized solution for children's physical and mental health development for children's health diagnosis and intellectual development, and improve their living habits and lifestyle (12). When training infant language through machine learning, we should combine language with knowing concrete things. It is necessary to combine language teaching with specific activities, such as pointing to apples and saying “This is an apple” when giving children apples, and knowing the specific thing of apples while teaching the pronunciation of apples. Use machine learning theory to analyze and mine children's health diagnosis and intelligence development, establish a health state model, and intuitively show people the health status of children's physical and mental health development in the form of data (13, 14). Moreover, the continuous accumulation of these big data is very important in the scientific research in the field of medical and health driven by big data.

## Related Work

The study (15) suggests that early childhood is the top priority of all aspects of children's development, and the problems related to children's education during this period can't be ignored, especially the significance to children's development can't be underestimated. In the game, let children learn something, which has a profound impact on the development of children's intelligence. Early childhood is the most important period to enhance the intelligence level of children. Education in the game mode is one of the important methods for enhancing the intelligence development of children.

The study (16) suggests that through the method of big data analysis, the newborn is crying, and others can't make it quiet, while the mother is in her arms. When the newborn touches the mother's skin and hears the mother's heartbeat, it will be quiet. Parents and relatives often make intimate and affectionate skin contact with the newborn, which is not only conducive to the development of the child's body and mind, but also conducive to the development of the child's intelligence.

A literature (17) research shows that 40–72% of the abnormal data processing actions of children's health data processing are an empty processing program or only record processing. In the worst case, this lack of proper action will not only not solve the problem, but even also lead to the bad development of the situation.

The study (18) suggests that mothers show affectionate skin contact by hugging, kissing, and gently massaging newborn child, especially skin contact and early sucking within half an hour after birth. This can not only compensate for the lost close skin contact in the mother's body, but also extend and expand the feeling of comfort, so that the newborn can form a positive skin feeling conditioned reflex.

The study (19) shows that through the method of big data analysis, skin contact and massage is a physiological need of human beings and all warm-blooded animals. When child can't get this kind of need or satisfaction, skin hunger will happen, which will affect their psychological mood, normal psychological development, and intelligence development.

The study (20) shows that to improve the children's intellectual development, parents and teachers both need to drive children to participate in games, especially more games conducive to improving their intelligence, which cannot only make children have game experience, but also improve the children's subjective initiative and cognitive ability while playing games, so as to obtain intellectual development.

A study (21) suggests that the treatment of children's abnormal physical condition in kindergartens at this stage is still in the artificial stage, which is inefficient and takes a long time, which not only increases the workload of kindergarten staff, but also may delay the treatment of children's condition. The study (19) shows that through the big data analysis method, for kindergartens, today's preschool teachers choose to educate children. The contents that preschool teachers think are “interesting” and children are not easy to operate. In addition, they will place too much emphasis on learning in the classroom and ignore the diversity of learning styles. Therefore, this will not necessarily promote the development of children's intelligence.

Research (22) shows that after birth, because newborns love their mothers, when they come into contact with their mothers' skin, they will feel happy and have enough sense of security, which is also an important test for early newborn health care. Skin sensation is the earliest sensation in infants, followed by smell, taste, and hearing.

The study (23) proposes that children's information is uploaded to the cloud to update the database, and parents can view the children's information through mobile app. In 2017, Li Qingyi and song Xueliang disclosed an all-in-one kindergarten morning check-up and medical care machine, which proposed to integrate the kindergarten common disease check-up and nursing materials. Parents can view nursing suggestions through mobile app.

For the problem of infant health diagnosis and infant intelligence development, this research project proposes to study infant health diagnosis and intelligence development based on machine learning and health information statistics. Developing children's mental and physical health through learning and playing can improve the children's subjective initiative and help children's motivation, knowledge, and ability.

## Principle and Algorithm of Machine Learning

**Figure 1:**
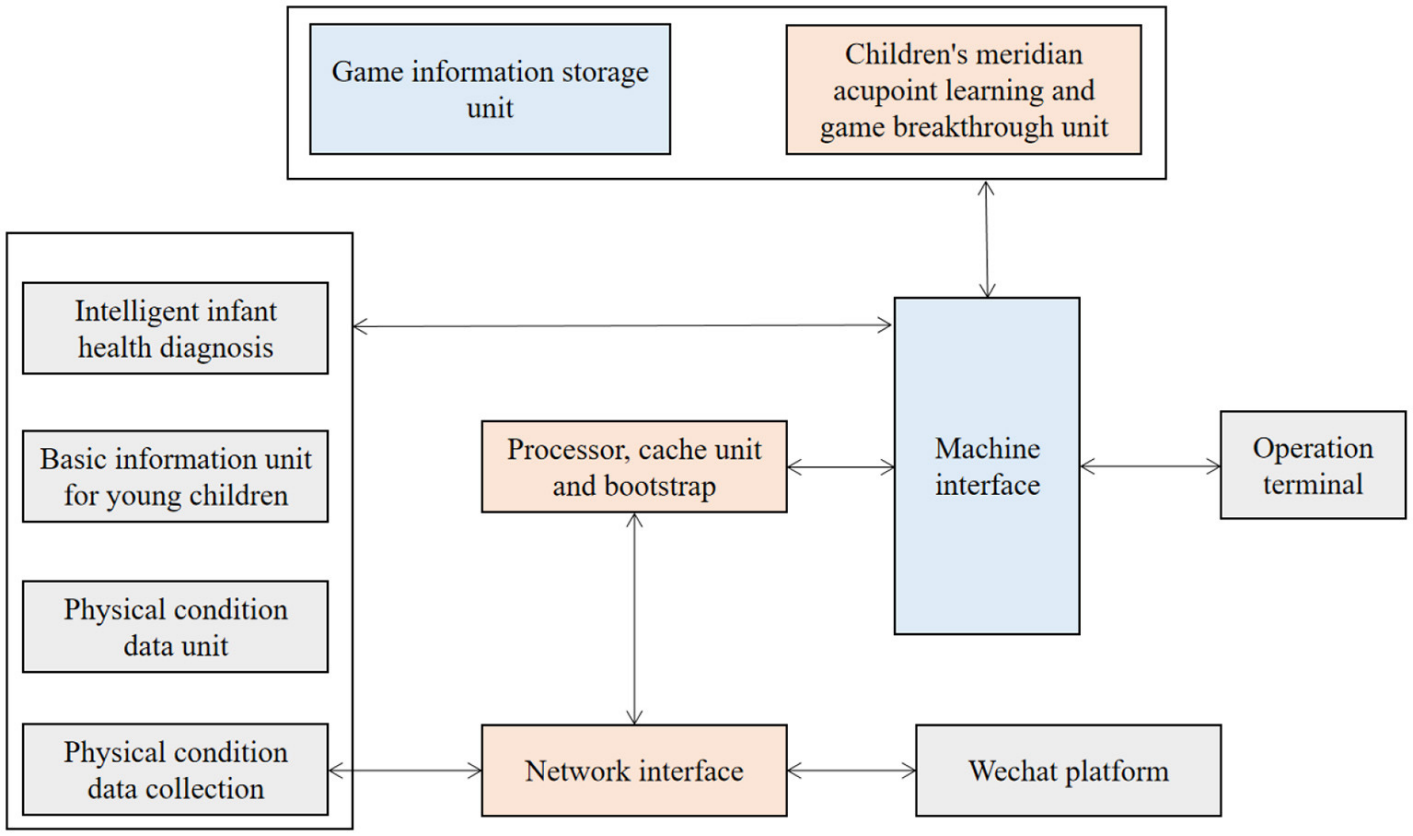
Schematic diagram of the structure of infant intelligent health diagnosis and intelligence development.

This topic integrates the automatic detection of children's physical and mental health development, the automatic management of children's information data and children's intelligence development. It has high degree of automation, strong reliability, and diversified functions, which is conducive to children's health and intelligence development. As shown in Figure 1.

The infant intelligent health diagnosis and intelligence development device comprises an operation terminal, an infant health self-test device, a processor, an infant intelligence development trainer, a computer interface, and a network interface. The operation terminal is connected with the processor through the network interface, and both the infant health self-test device and the infant intelligence development trainer are connected with the processor through the interface. The processor includes a processor and a storage unit, and the infant health self-test device and the infant intelligence development trainer, respectively, include the infant health self-test unit, the infant intelligence development training unit, and their respective boot programs and storage units.

Machine learning algorithm is used to construct the model of child health diagnosis and intelligence development. When using machine learning to solve problems, logistic regression is usually the first choice and then other complex algorithms are used on this basis. In order to solve the classification problem, firstly, the sigmoid function is used to construct the prediction function, that is, the classification task. Then, the *J*(θ) function is derived from the maximum likelihood estimation. Finally, the optimal model coefficients are solved by the gradient descent algorithm.

The function form is as follows:


(1) $$\begin{array}{l} g\left(z\right)=\frac{1}{1+e^{-z}} \end{array}$$

The independent variable z is any real number, and the range is [0,1]. That is, the conversion from numerical value to probability is completed by sigmoid function, and the prediction function is as follows:


(2) $$\begin{array}{l} h_{\theta }\left(x\right)=g\left(\theta^{T}x\right)\frac{1}{1+e^{-\theta^{t}x}} \end{array}$$

where x is the dataset introduced in the form of matrix, $\theta_{0}x_{0}+\theta_{1}x_{1}+......+\theta_{n}x_{n}=\overset{0}{\underset{i=0}{\sum }}\theta_{i}x_{i}=\theta^{T}x$, and the best parameter is $\theta ={\left[\theta_{0},\theta_{1},\ldots \theta_{n}\right]}^{T}$.

For the binary classification task (0,1), the prediction function is constructed as follows:


(3) $$\begin{array}{l} P\left(y=1|x,\theta \right)h_{\theta }\left(x\right),P\left(y=0|x.\theta \right)=1-h_{\theta }\left(x\right) \end{array}$$

For the sake of simplicity, the two-category tasks can also be integrated into


(4) $$\begin{array}{l} p\left(y|x;\theta \right)={\left(h_{\theta }\left(x\right)\right)}^{y}{\left(1-h_{\theta }\left(x\right)\right)}^{1-y} \end{array}$$

Based on the prediction function, the maximum likelihood estimation *J*(θ) function is constructed as follows:


(5) $$\begin{array}{l} J\left(\theta \right)=\frac{1}{m}\log p\left(y|x,\theta \right)=-\frac{1}{m}\overset{m}{\underset{i=1}{\sum }}\left(y_{i}\log h_{\theta }\left(x_{i}\right)+\left(1-y_{i}\right)\log\left(1-h_{\theta }\left(x_{i}\right)\right)\right) \end{array}$$

*J*(θ) converting the gradient ascending task into the gradient descending task.

The optimal coefficient $\theta_{j}^{*}$ can be obtained by solving the first-order equation of *J*(θ) to θ_*j*_.


(6) $$\begin{array}{l} \frac{\partial J\left(\theta \right)}{\partial \theta_{j}}=\frac{1}{m}\overset{m}{\underset{i=1}{\sum }}\left(h_{\theta }\left(x_{i}\right)-y_{i}\right)x_{i}^{j} \end{array}$$

where $x_{i}^{j}$ represents the sample value of row I and column J.

Finally, the optimal solution is obtained by parameter updating.


(7) $$\begin{array}{l} \theta_{j}^{*}=\theta_{j}-\alpha \frac{1}{m}\overset{m}{\underset{i=1}{\sum }}\left(h_{\theta }\left(x_{i}\right)-y_{i}\right)x_{i}^{j} \end{array}$$

The logistic regression requires simple input data of the model. The feature set can be classified variables or continuous variables, and the modeling process is also convenient. Therefore, it is a widely used classification model. In the field of disease prediction, unconditional logistic regression model is widely used.

Let the separation hyperplane separate the linearly separable sample set *T* = {(*x*_*i*_, *y*_*i*_), *i* = 1, ..., *N*} correctly, that is


(8) $$\begin{array}{l} y_{i}=\{\begin{array}{cc} 1 & w\cdot x_{i}+b\geq 1\\ 0 & w\cdot x_{i}+b\leq 1 \end{array} \end{array}$$

The classification interval of the separation hyperplane is defined as


(9) $$\begin{array}{l} d=d_{+}+d_{-}=\frac{\left|w\cdot x+b\right|}{\left||w|\right|}=\frac{1}{\left||w|\right|} \end{array}$$

Among


(10) $$\begin{array}{l} d_{+}=\underset{i:y=1}{\min}\left\{\left(w\cdot x_{i}+b\right)/||w||\right\} \end{array}$$

(11) $$\begin{array}{l} d_{-}=\underset{i:y=-1}{\min}\left\{\left(w\cdot x_{i}+b\right)/||w||\right\} \end{array}$$

Indicates the distance between the sample points and the decision surface equation. To maximize the hyperplane classification interval d, it can be transformed into a set of w &b to minimize ||*w*||. For the later calculation aspect, it is transformed into a set of w & b, which minimizes ||*w*||^2^. When the similarity between the collected characteristic information of infant's physical condition and a disease in the case information database is 0, the infant does not have the disease; when the similarity is not >0.4, the infant's physical condition is sub-healthy for the disease; when the similarity is >0.4, the infant is suspected to have the disease. Judgment of infant's physical health or suspected illness, when the similarity between the collected infant's physical condition characteristic information and all the illnesses in the case information base is 0, the infant is healthy; If the similarity of all diseases is not <0, i and not more than 0.4, the child's body is in a subhealth state; If the similarity of all diseases is not <0.4, the infant suffers from diseases, and the disease with higher similarity value is taken as the preliminary diagnosis result.

**Figure 2:**
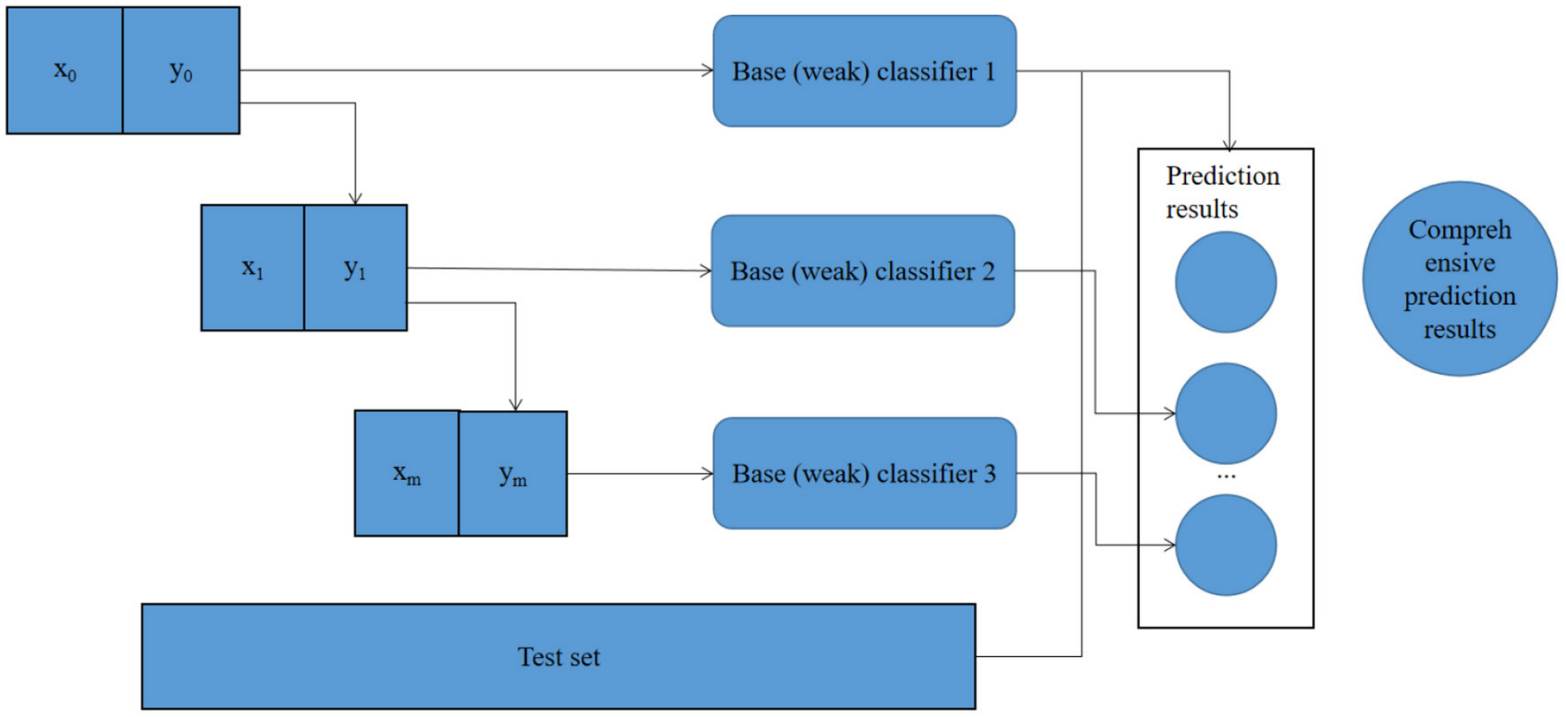
Training flow chart of logistic regression algorithm model.

Different machine learning algorithms are obtained due to different function selection. However, the training process of logistic regression algorithm is to train each weak classifier in step form, and then combine the weak classifiers into strong classifiers in a certain way to obtain the comprehensive prediction results, as shown in Figure 2.

Considering the model principle and the data characteristics of this study, the logistic regression algorithm in machine learning algorithm will be used to construct the model of infant health diagnosis and intelligence development, and to find the most suitable model for infant health diagnosis and intelligence development.

## Infant Health and Intelligence Development

### Intelligent Diagnosis and Disease Treatment

#### Identification of Young Children

With the development of information technology, there are more and more forms of identification technology, which can be summarized into the following three categories: the first category is the things you can know, such as passwords, which are easy to crack and have the lowest relative security; The second category is the things you can own, such as smart cards, which are not easy to save, easy to lose, and low in security. The third category is the personal characteristics held by everyone, such as face and iris. These characteristics are unique to everyone. Compared with the first two categories, the third category is more trustworthy. Ensure the nutrition requirement of the first rapid proliferation of brain cells in the fetal period, and increase the number and quality of brain cells as much as possible with sufficient and high-quality nutrition. Pregnant women should pay attention to strengthening nutrition, eating reasonably and supplementing folic acid and trace elements such as zinc, iron, and copper. Iodine deficiency leads to dementia, and iodine deficiency areas should pay special attention to iodine supplementation. The important role of early education can't be replaced and compensated by any other form of education in the future. Long-term emotional stress and anxiety of pregnant women, fright, fear, sadness, high-dose radiation, organic mercury, lead and other toxic substances, tobacco and alcohol, virus infection, pregnancy poisoning, diabetes and hypothyroidism, taking phenytoin sodium, antithyroid drugs, perinatal hypoxia, and birth injury, etc. Grasp the “best age,” that is, it is easier to learn certain knowledge or behavior at a certain age, faster to master and better to remember, and to carry out education in advance or delay, and the effect is far less than the best age. For babies, the facial movements should be trained first. Modern scientific research has proved that the more and more complex fingers move, the more they stimulate the brain, thus promoting the development of intelligence, so we should pay attention to the training of child's facial movements.

The specific steps of children's face training or recognition algorithm are as follows:

① Connect the camera.

② Camera device captures children's faces.

③ Extract and mark the captured children's faces.

④ For face training, name the captured child's face and store it in the child information database for training. For face recognition, compare the captured child's face with the child's face in the child information database. If the comparison is successful, extract the child's student status number, name, and other information; otherwise if you choose to continue, carry out infant face training. If you choose to end, carry out the next step.

⑤ End.

#### Children's Physical Condition Information Collection

Children's physical condition information collection is the premise of intelligent diagnosis of children's physical health. Its collection includes temperature acquisition, cough, diarrhea, runny nose, and so on. Genetically speaking, the development of human brain is influenced by genes and environment. The structure and function of brain are directly related to the quality of genes, but also influenced by environmental factors. Brain development is a process that determines the gradual expression of genes in the brain. In the process of gene expression, unfavorable or even harmful environmental factors can inhibit gene expression, causing brain development disorder or damage, while appropriate and favorable environmental factors can effectively promote gene expression, making the brain structure and function develop fully and perfectly. Early education is the most effective environmental stimulation factor to promote brain development, which can fundamentally improve the fine structure and function of brain, make it fully developed and perfect, not only improve the development quality of normal brain, but also significantly improve the intelligence level of mentally retarded people. Emphasize the nutritional conditions and further improve the quality of brain cells. Breast milk is the most ideal natural food, and breastfeeding is the most concerned issue in all countries today. The WHO requires that babies should be breastfed for at least 4 months after birth, just to meet the nutrition needs of the second rapid proliferation of brain cells. There are some differences in the reports about the influence of diet on physical development in different places. In some places, it is reported that children are eating too much animal protein at present; in some areas, children are eating too much plant food, and the proportion of nutrition intake among children in different areas is also different.

Strengthen the healthcare of infants' physical and mental health development. Infant immune system is imperfect and prone to diseases. Many diseases affect intellectual development. Therefore, active prevention and treatment should be carried out to minimize the occurrence of diseases and shorten the course of disease, so as to ensure the healthy development of the brain. In the past, when talking about the impact of diet on children's physical development, people immediately thought of malnutrition, which is indeed a very important aspect. How to ensure the healthy growth and development of children on the basis of solving the problem of malnutrition? There are many literature reports that with the improvement of material living standards, there are many new problems in children's physical development in China, such as children's obesity, anemia, precocity, physical decline, and so on. The non-contact infrared thermometer is used to obtain the temperature of children, which is wirelessly sent to the wireless receiver and stored in the collection unit of children's physical and mental health development. The utility model has the advantages of high accuracy of the measured body temperature. After obtaining the temperature of children, analyze whether the temperature is normal based on medical knowledge, and analyze the degree of fever for abnormal temperature. Biological studies have confirmed that the diet structure is closely related to children's physical and psychological growth and development, and is related to the occurrence of many diseases. In this paper, based on machine learning method, children's identity is recognized by automatically collected physical condition data and faces, and children's health status is diagnosed by case-based reasoning by using physical condition data of children. However, only for sick children, the treatment scheme of over-the-counter standing drugs is given by man–machine combination according to their severity. Therefore, on the basis of the proposed training method of intelligent infant health diagnosis and intelligence development, this paper designs an intelligent infant health diagnosis and intelligence development device. This research device has strong reliability and diversified functions, which are beneficial to the infant's health and intelligence development, but there are still some areas that need further research. Even people's personality characteristics, ability tendency and intelligence level are also related to diet. Chinese children's unreasonable diet has various manifestations. According to the literature, the main reason is that the food is too delicate and destroys the content of vitamins and minerals of the food. The relationship between unreasonable dietary structure, nutritional imbalance, and intellectual disability has been recognized by the society, such as iodine deficiency and intellectual disability, and the impact of zinc deficiency on intelligence. However, how to make children with normal development have better intelligence has not attracted extensive attention from the society. The children's perception and cognitive ability, as well as the flexibility and accuracy of children's hands, are trained through three links: self-study of human meridians and acupoints, chapter test, and game breakthrough. Comparative analysis shows that the proposed method can integrate learning, entertainment, and healthcare, and play a role in enhancing the physical and mental health of children and also in developing the children's intelligence.

### Results and Analysis

After the original data are processed by SMOTE algorithm, the new dataset is modeled by the same method and its performance is compared. The recall rate, f-score, accuracy, and Area under curve (AUC) of the random forest model are higher than those of the other two models, and the specific results are shown in Tables 1, 2.

**Table 1:** The comparison of the performance of data after three classification models applied to health information statistics for children's health diagnosis and intelligence development.

**Model**	**Recall**	**Accuracy**	**F-score**	**A**	**AUC**
				**Accuracy**	
Logistic	0.602	0.935	0.733	0.813	0.895
Random	0.735	0.907	0.812	0.855	0.943
forest					
SVM	0.735	0.889	0.804	0.848	0.896

**Table 2:** The performance comparison of data processed by health diagnosis and intelligence development of children with three classification models applied to health information statistics.

**Model**	**Recall**	**Accuracy**	**F-score**	**Accuracy**	**AUC**
Logistic	0.605	0.931	0.730	0.816	0.892
Random	0.738	0.913	0.815	0.857	0.947
forest					
SVM	0.736	0.892	0.814	0.847	0.899

Through the investigation of kindergarten a, the specific results of children's physical condition are shown in Table 3.

**Table 3:** Comparison of health diagnosis and intelligence development of children in kindergarten a.

**Factor**	**Number of**	**Number of**	**Rate (%)**	**X2 value**	***P* value**
	**people**	**people**			
	**surveyed**				
Small class	156	58	37	1.508	0.221
Middle shift	168	67	40	3.36	0.065
Taipan	197	89	45	4.571	0.064

**Figure 3:**
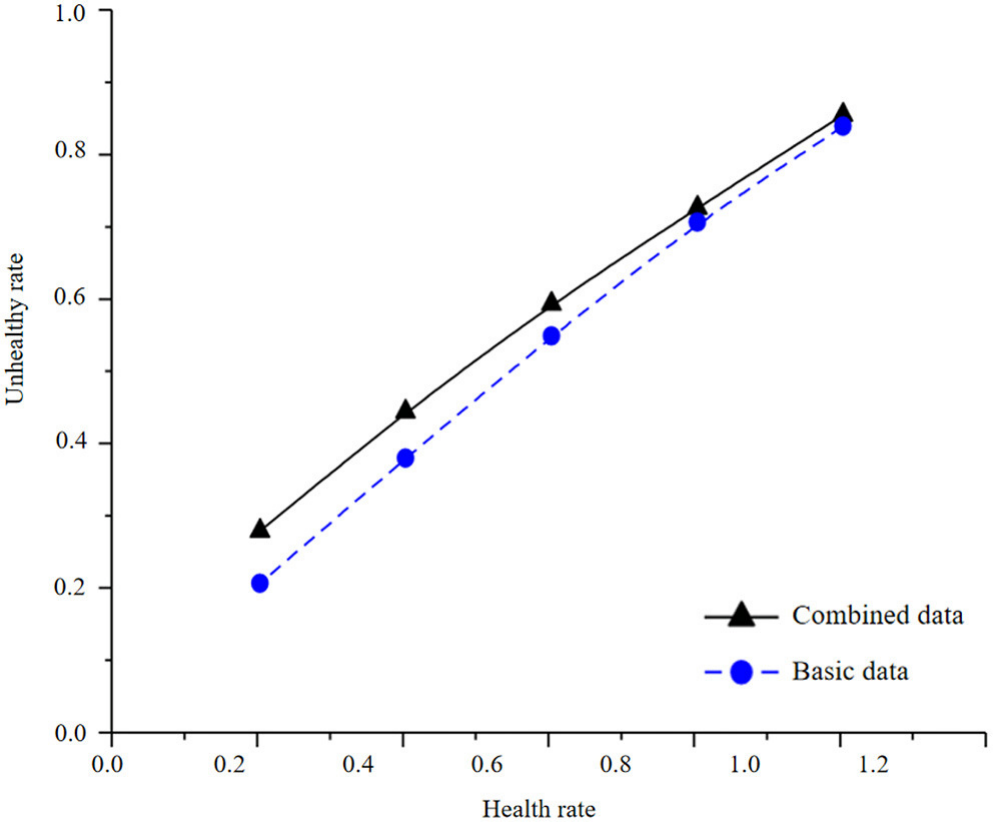
Health information statistics of logistic regression combined data and basic data.

**Figure 4:**
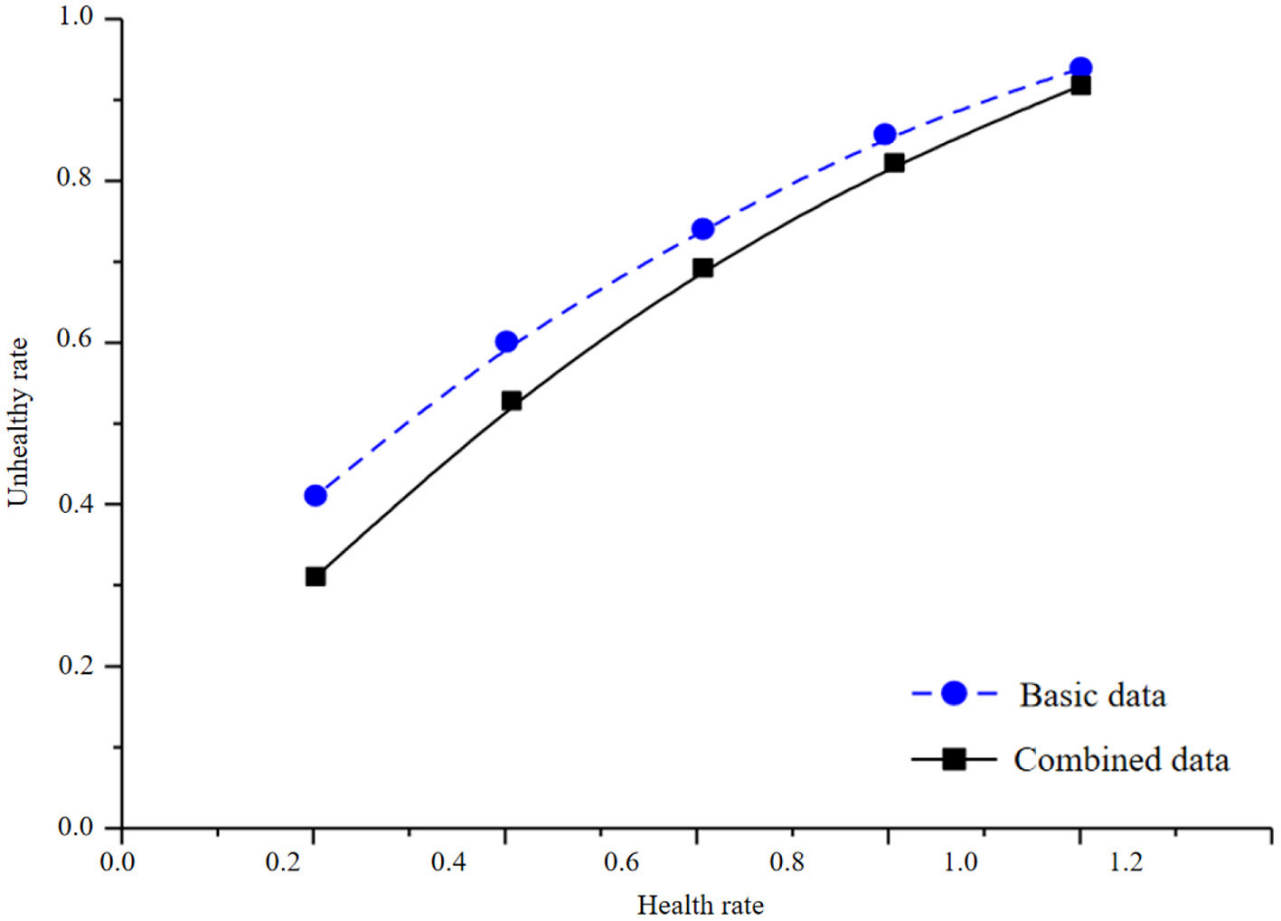
Health information statistics of logistic regression combined data and basic data.

In the process of infant health diagnosis and intelligence development based on machine learning and health information statistics, the accuracy, precision, recalls rate, F1 value, and AUC value are used to evaluate the effects of basic data model and combined data model. By constructing Logistic regression model, the results are shown in Table 4 and Figures 3, 4.

**Table 4:** Logistic regression model evaluation of basic data and combined data.

**Input data**	**Data set**	**Health information statistics**				
		**Accuracy**	**Accuracy**	**Recall**	**F1 value**	**AUC**
Basic data	Training set	74.39	75.78	73.47	74.60	82.24
	Test set	74.41	75.05	73.05	74.04	81.33
Combined data	Training set	74.91	75.60	75.28	75.45	84.18
	Test set	77.32	78.62	74.98	76.75	83.66

By comparing the modeling results of basic data and combined data, it is found that there is an obvious gap between the modeling results of basic data and combined data. The AUC of the test set of combined data is 83.66%, and that of the test set of basic data is 81.33%. In logistic regression modeling, the AUC of the combined data model is 2.33% higher than that of the basic data, and the accuracy, accuracy, recall F1 values are also greatly improved, indicating that the precision of the model is also improving accordingly. It can be seen from the observation figure that the gap between the AUC results of the training set and the test set is small in the combined data and the basic data, and there is no obvious gap between the accuracy, recall, and F1 value of the training set and the test set of the combined data and the basic data, indicating that there is no overfitting phenomenon in the model.

**Figure 5:**
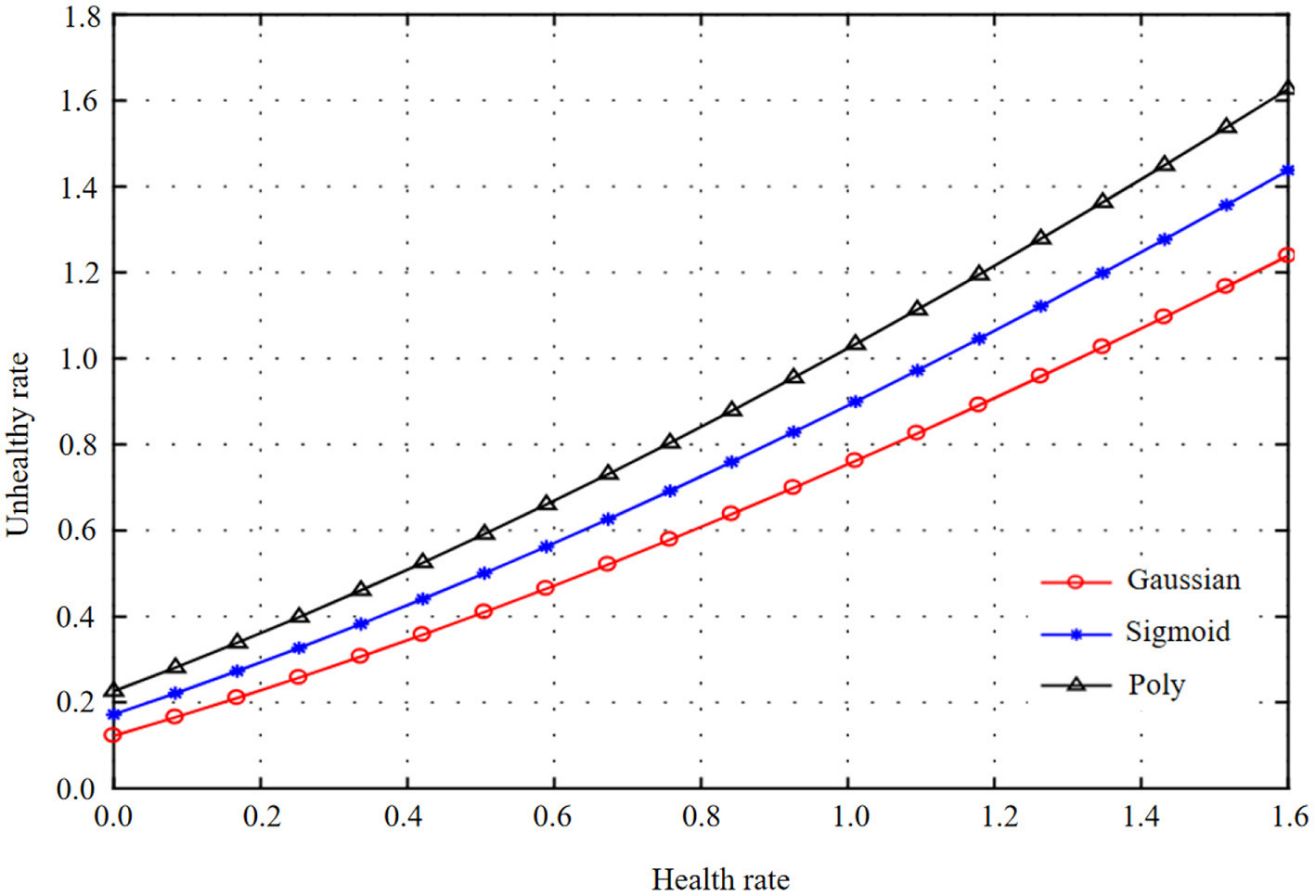
Curve representations of three kernel functions of machine learning basic data and health information statistics.

**Figure 6:**
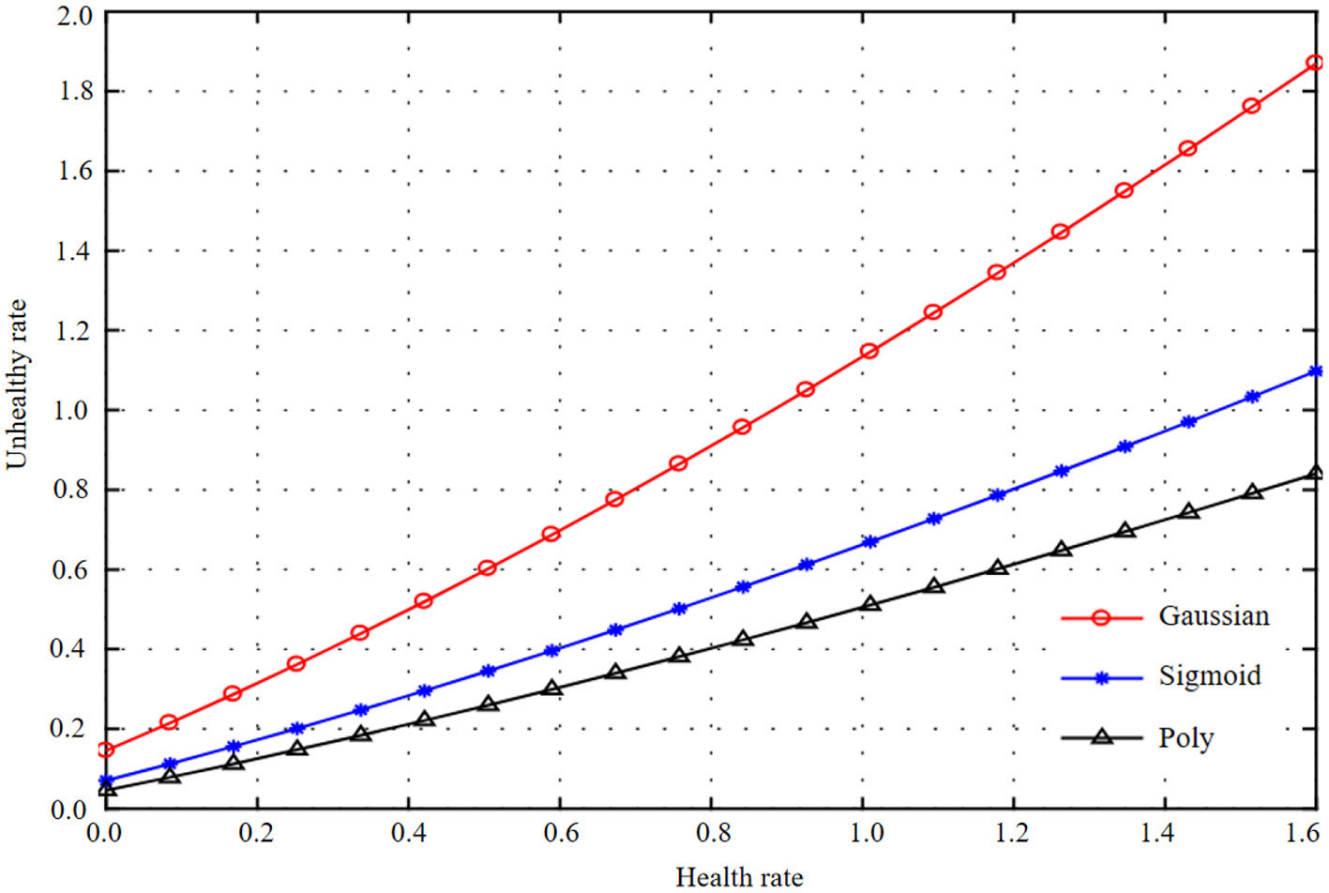
Curve representations of three kernel functions of machine learning basic data and health information statistics.

**Figure 7:**
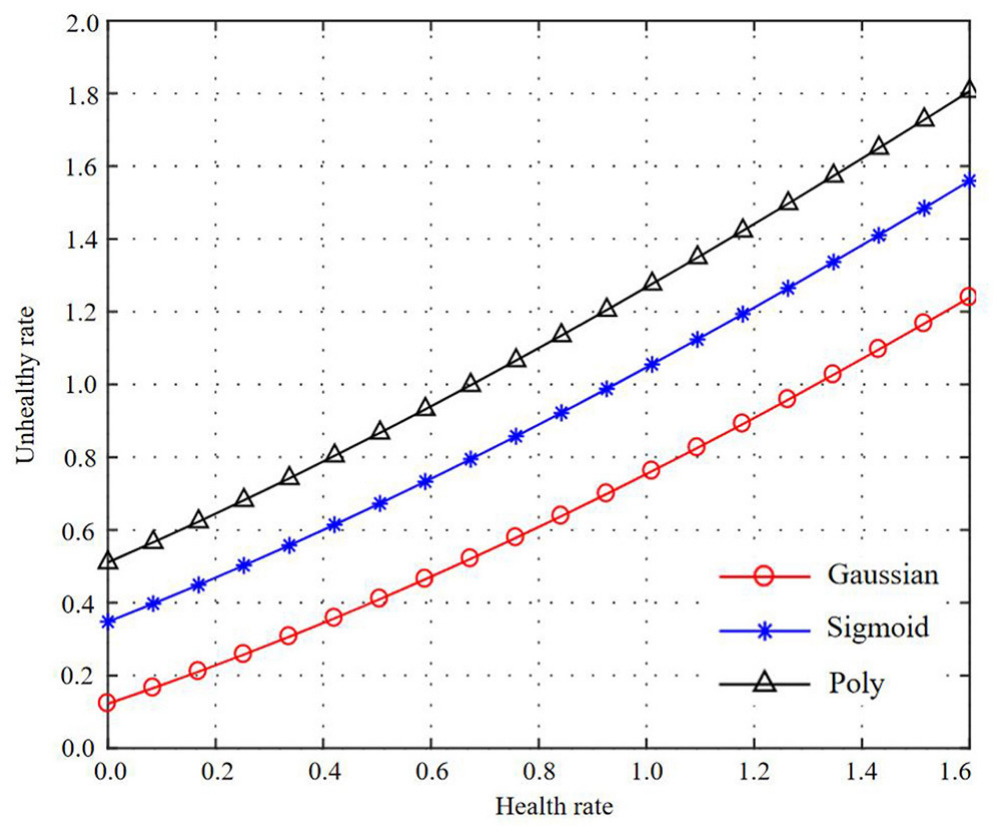
Curve representation of three kernel functions of machine learning basic data and health information statistics.

Using basic data for modeling, the optimal parameters and model evaluation results of different kernel functions are obtained. Get Working characteristic curve (ROC) curves of different kernel function test sets and training sets, as shown in Figures 5–7. At the same time, the accuracy, precision, recall rate, F1 value, and AUC value of each model are shown in Table 5.

**Table 5:** Optimal parameter combination of different kernel functions modeling results of machine learning basic data and health information statistics.

**Optimal parameter combination of different kernel functions**	**Data set**	**Health information statistics**				
		**Accuracy rate**	**Precision**	**Recall rate**	**F1 value**	**AUC**
Ploy kernel function	Training set	72.78	86.87	55.17	67.45	84.18
C = 0.1, degree = 3 gamma = 0.01	Test set	71.63	83.72	53.63	65.37	80.45
Gaussian kernel function	Training set	76.63	78.72	74.48	76.54	84.28
C = 1, gamma = 0.01	Test set	76.23	77.68	73.53	75.55	82.57
Sigmoid kernel function	Training set	73.14	75.15	71.03	73.03	79.98
C = 0.1, gamma = 0.1	Test set	73.44	74.54	71.11	72.78	80.73

As can be seen from Figures 5–7, the test set accuracy of Gaussian kernel function is 74.54%, which is <83.72% of polynomial kernel function, but the recall rate and F1 value of polynomial kernel function are low, indicating that although polynomial kernel function ensures the model precision, the recall rate is low. In addition to the accuracy, other indexes of Gaussian kernel function are the best; especially the AUC of the test set of Gaussian kernel function is 82.57%, which is the largest among all kernel functions. The gap between the training set and the test set of the three SVM kernel functions is small, indicating that there is no fitting phenomenon in the training process. Through comprehensive comparison and analysis, the Gaussian kernel SVM model with C = 1 and gamma = 0.01 is finally selected as the best model for basic data modeling.

**Figure 8:**
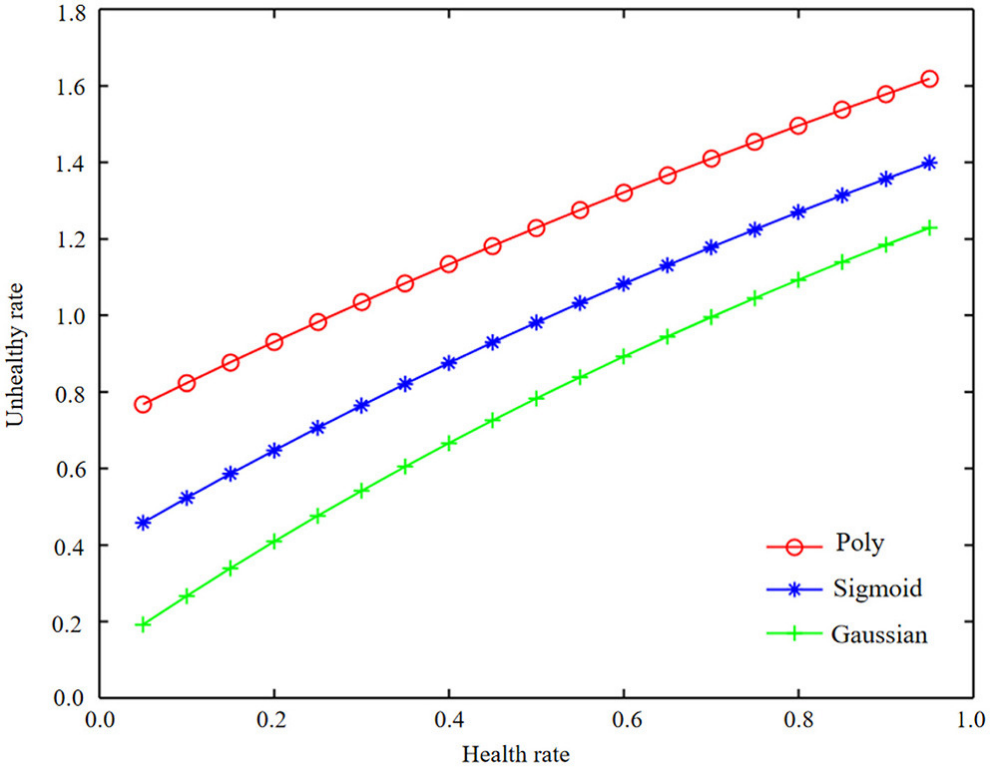
The ROC curve representation of three kernel functions under machine learning combined data.

**Figure 9:**
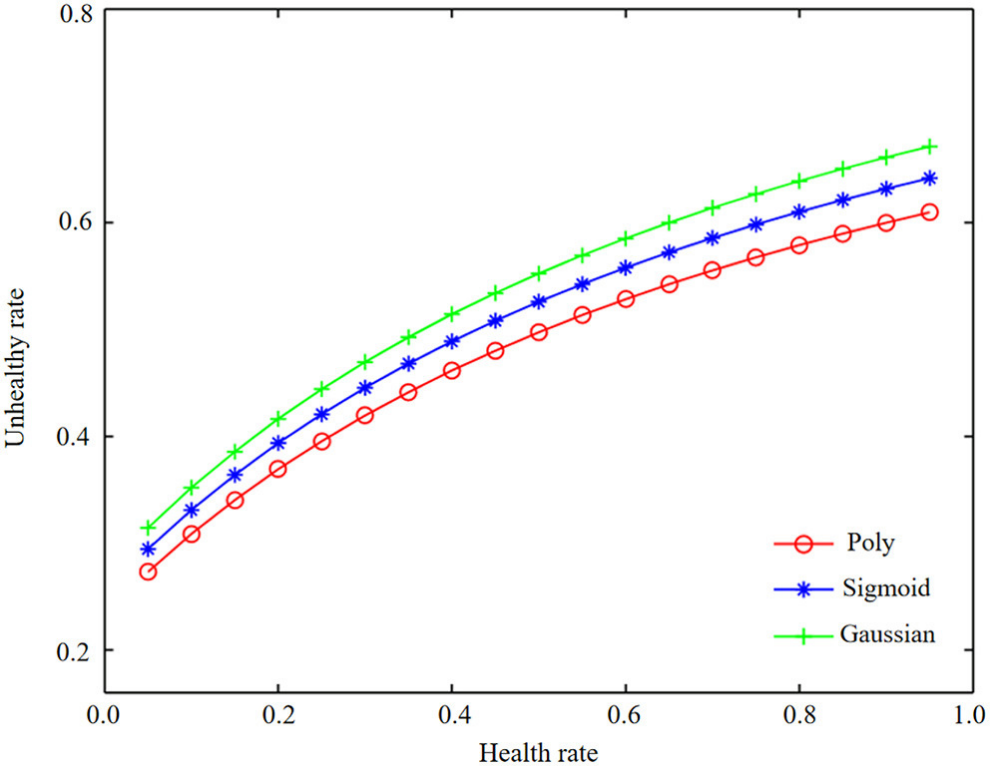
The ROC curve representation of three kernel functions under machine learning combined data.

**Figure 10:**
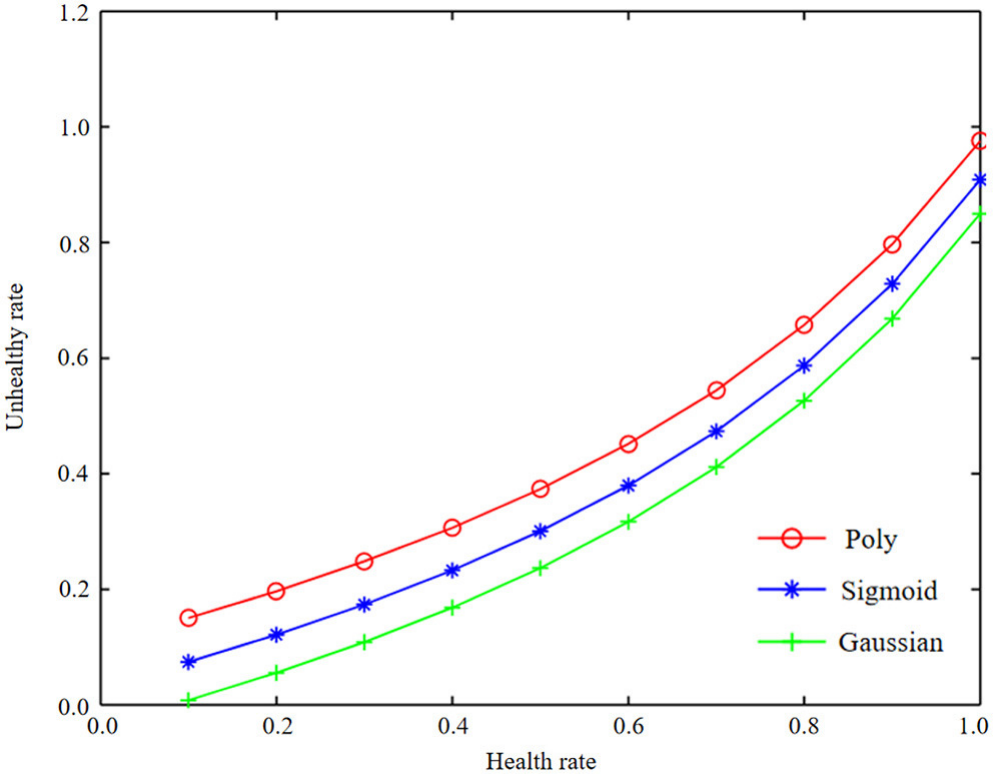
ROC curve performance of three kernel functions under machine learning combined data.

Using the combined data to model different kernel functions of SVM, the optimal parameters and model evaluation results of different kernel functions are obtained. The values of accuracy, precision, recall, F1, and AUC are shown in Table 6. Meanwhile, ROC curves of different kernel functions are shown in Figures 8–10.

**Table 6:** Modeling results of different kernel functions of SVM under machine learning combined data.

**Optimal parameter combination of different kernel functions**	**Data set**	**Health information statistics**				
		**Accuracy rate**	**Precision**	**Recall rate**	**F1 value**	**AUC**
Ploy kernel function	Training set	83.76	88.52	78.45	83.18	93.05
C = 0.1, degree = 3 gamma = 0.01	Test set	76.11	80.44	68.92	74.24	82.31
Gaussian kernel function	Training set	78.12	79.22	77.53	78.37	86.52
C = 1, gamma = 0.01	Test set	77.32	78.91	73.50	76.64	83.76
Sigmoid kernel function	Training set	73.40	78.91	64.83	71.21	81.53
C = 0.1, gamma = 0.1	Test set	75.38	78.44	66.51	72.96	82.45

As can be seen from Figures 8–10, in the machine learning algorithm, the accuracy of Gaussian kernel function is slightly less than polynomial kernel function and Sigmoid kernel function, but other indexes are the best, and the gap between the test set and training set of SVM model of Gaussian kernel function is small, which shows that the model has hardly been fitted. According to the comprehensive comparison of index accuracy, precision, recall rate, F1 value, AUC value, and fitting effect, we finally choose the Gaussian kernel SVM model with C = 1 and gamma = 0.01 as the best model for combined data modeling.

## Conclusions

Children's health level and intellectual development level are the basis of children's other aspects. Although the current products can carry out children's morning examination and daily management of kindergartens, with the development of modern information technology, it is necessary to design a product to integrate children's health examination and intellectual development, as well as learning and entertainment. Based on the training methods of intelligent children's health diagnosis and intelligence development based on machine learning and health information statistics, this paper designs children's intelligent health diagnosis and intelligence development device. The research device of this subject has strong reliability and diversified functions, which is conducive to children's physical health and intelligence development, but there are also places that need to be further studied. Based on machine learning, several basic learners are integrated to train and predict the data, and the prediction effect is usually better than the traditional machine learning model. The method of intelligent infant health diagnosis and man-machine combination for infant disease treatment is proposed. This method recognizes the identity of children through the automatically collected physical condition data and face, uses the child's physical condition data to diagnose the child's health condition based on case-based reasoning, gives the treatment scheme of over-the-counter standing drugs for the sick children according to their severity, and publishes the diagnosis results and treatment suggestions to parents through WeChat platform.

## Data Availability Statement

The original contributions presented in the study are included in the article/supplementary material, further inquiries can be directed to the corresponding author.

## Ethics Statement

The studies involving human participants were reviewed and approved by Chengdu University. Written informed consent for participation was not required for this study in accordance with the national legislation and the institutional requirements.

## Author Contributions

SW and ML: conceptualization, methodology, validation, resources, data curation, writing review, and editing. SW: software, formal analysis, investigation, writing original draft preparation, project administration, and funding acquisition. ML: visualization and supervision. All authors have read and agreed to the publishing version of the manuscript.

## Funding

This work was supported by the Center for Early Childhood Educatoon Research of Sichuan, China (CECER-2020-B03); Social Science Planning Office of Chengdu, China (YN2420210747).

## Conflict of Interest

The authors declare that the research was conducted in the absence of any commercial or financial relationships that could be construed as a potential conflict of interest.

## Publisher's Note

All claims expressed in this article are solely those of the authors and do not necessarily represent those of their affiliated organizations, or those of the publisher, the editors and the reviewers. Any product that may be evaluated in this article, or claim that may be made by its manufacturer, is not guaranteed or endorsed by the publisher.
